# Moderate leg length discrepancy: a long-term risk factor for hip osteoarthritis?

**DOI:** 10.1186/s10195-026-00909-7

**Published:** 2026-03-23

**Authors:** Alessandro Aprato, Andrea Donis, Riccardo Giai Via, Andrea Scandurra, Francesco Tuè, Andrea Audisio, Federico Fusini, Alessandro Massè

**Affiliations:** 1 Department of Surgical Sciences, University of Turin, Viale 25 Aprile 137 Int 6, Turin, Italy; 2 Trauma and Orthopaedic Centre, Città della Salute e della Scienza, Turin, Italy

**Keywords:** Leg length discrepancy, LLD, Limb dysmetria, Hip osteoarthritis, Hip joint degeneration, Biomechanics

## Abstract

**Background:**

Leg length discrepancy (LLD) has been implicated as a biomechanical factor contributing to hip osteoarthritis (OA), yet the extent of its influence remains unclear. This study examines the correlation between moderate LLD (≥ 10 mm) and hip OA progression, focusing on asymmetrical OA distribution in patients over 65 years old.

**Materials and methods:**

A retrospective analysis was conducted from a database of 1672 full-length standing X-rays. Patients under 65 years, with deformities, unilateral limb issues, or prosthetics, were excluded; therefore, the study group was composed of 220 patients. Tibial and femoral lengths were measured bilaterally, and hip OA severity was assessed using the Tönnis classification. Statistical analyses included Pearson’s Chi-squared test and linear regression to explore correlations between LLD and OA distribution.

**Results:**

Among the sample, 18% showed an LLD ≥ 10 mm. A significant correlation was found between LLD and the asymmetrical distribution of hip OA (*p* = 0.002), with higher OA severity observed in the hypometric limb. Linear regression analysis suggested that each millimeter of LLD corresponded to a 0.74-unit change in OA severity difference between hips.

**Conclusions:**

This study highlights a significant association between moderate LLD and contralateral hip OA in the elderly, emphasizing the biomechanical impact of asymmetrical joint loading. Findings suggest the need for early identification and targeted management of LLD to mitigate OA progression.

**Level of evidence:**

III.

## Introduction

Lower limb length discrepancy (LLD) is a common condition encountered in daily clinical practice, presenting with a wide spectrum of clinical manifestations ranging from asymptomatic to awkwardness of gait, joint discomfort, and development of arthritis-related joint pain, etc. [1]. According to Knutson et al. [2] approximately 90% of the population presents a limb length discrepancy of at least 1 mm, while only 10% have perfectly equal limb lengths. In addition, about 50% of the population exhibits a discrepancy of 4 mm or less, and 90% have a discrepancy of 10 mm or less [3].

Some studies have associated lower limb length discrepancy as a predisposing factor for the development of hip osteoarthritis (OA), using different cut-offs and evaluation methods, obtaining results that are not always in agreement [4–8].

Individuals with LLD often adapt their movement patterns to functionally compensate for the discrepancy, such as increasing knee flexion or hip adduction on the longer limb [9, 10].

However, these compensatory strategies may amplify forces across a reduced joint contact area [11, 12], potentially serving as a biomechanical precursor to lower extremity issues [6, 13].

LLD leads to compensatory pelvic obliquity, altered lumbar spine alignment, and asymmetrical muscle activation during walking, mainly affecting the coronal plane alignment. These adaptations modify joint reaction forces and spinopelvic dynamics, increasing mechanical stress on the hip and lumbar spine [8, 14].

The aim of the study is to determine whether a limb length difference of 1 cm or more represents a significant cutoff for increased risk of OA progression.

In addition, the study aims to investigate how LLD contributes to the asymmetric distribution of OA by examining whether the condition predisposes individuals to greater joint degeneration in the contralateral hip of the longer limb or in the ipsilateral hip of the shorter limb. This analysis aims to clarify the different biomechanical and degenerative effects of LLD on both sides of the body. Furthermore, the research seeks to quantify the severity and distribution of OA in relation to the various degrees of LLD.

## Materials and methods

This retrospective study examined full-length standing radiographs archived and taken in a major teaching hospital from 1 January 2020 to 31 December 2024. All the full-length standing radiographs were reviewed by an orthopedics and traumatology resident and, in cases of uncertainty, re-evaluated by a highly experienced surgeon.

Only patients aged 65 years and older were included to focus on an age group more likely to have degenerative changes in the hip joint. Patients younger than 65 years were therefore excluded. This age cutoff was chosen to align the study with the objective of studying the progression of osteoarthritis [15].

Other exclusion criteria included X-rays with technical deficiencies, such as inadequate patient positioning suggestive of rotational malalignment or incomplete visualization of lower limb structures. Patients with limb deformities resulting from previous femoral and/or tibial fractures, treated either surgically or conservatively, were excluded to avoid potential measurement bias.

The presence of hip and/or knee replacements or surgical fixation devices also led to exclusion, as these implants could interfere with accurate radiographic evaluations and axis determination. By excluding these cases, the study ensured that measurements reflected native anatomical LLD rather than acquired LLD.

**Fig. 1:**
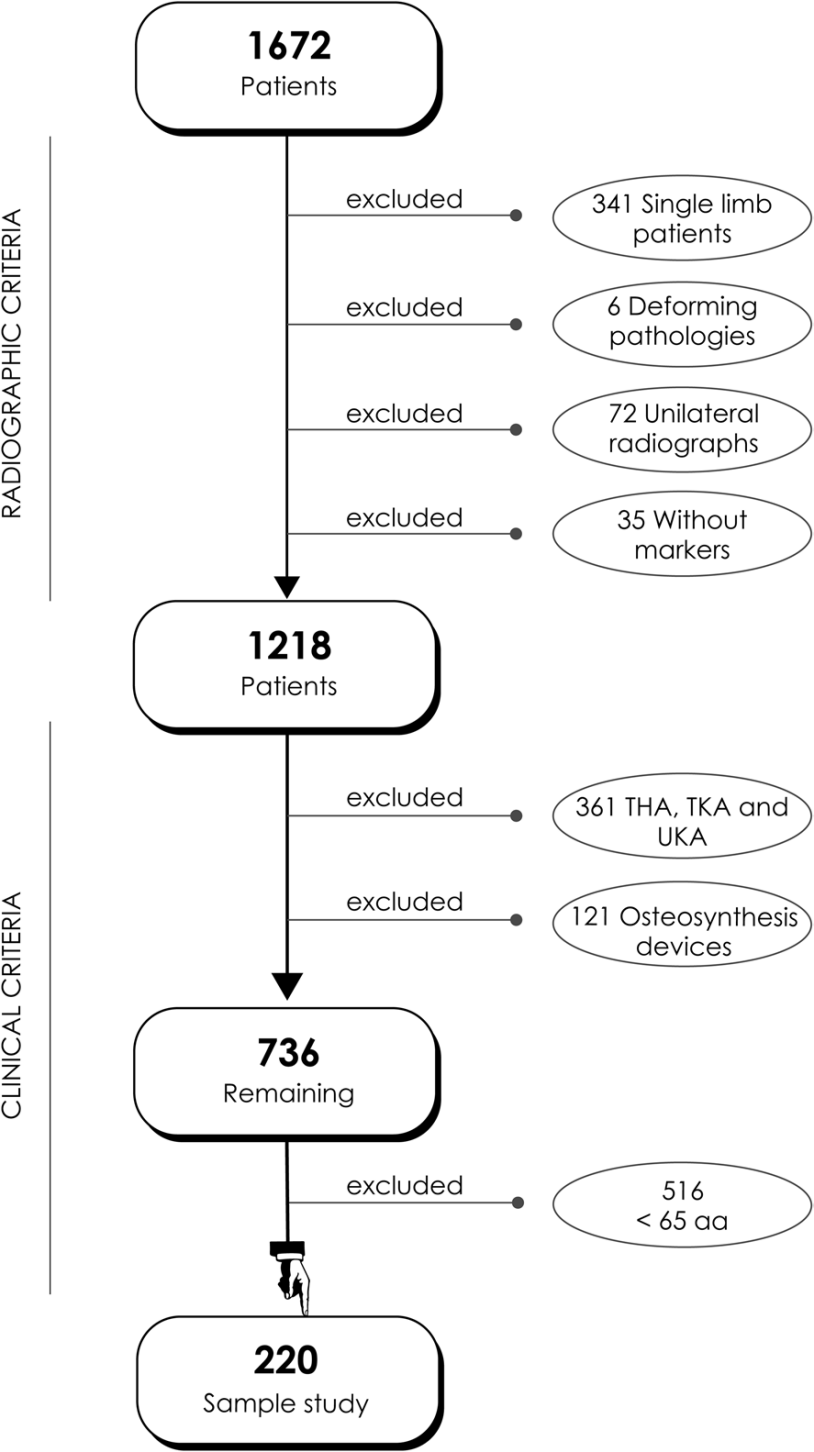
Flowchart showing the inclusion and exclusion criteria

A detailed flow chart illustrating the inclusion and exclusion criteria is shown in Fig. 1.

To assess the relationship between lower limb length discrepancy (LLD) and hip osteoarthritis (OA), precise measurements were obtained from full-length standing radiographs. Tibial length was measured bilaterally, using the intercondylar eminence as the proximal landmark and the center of the tibio-tarsal joint as the distal landmark. Similarly, the length of the femur was assessed from the greater trochanter to the center of femoral rotation (CFR), and from the greater trochanter to the intercondylar notch. These measurements provided comprehensive data on the structure of the lower limb, allowing a detailed analysis of any asymmetry [16].

**Fig. 2:**
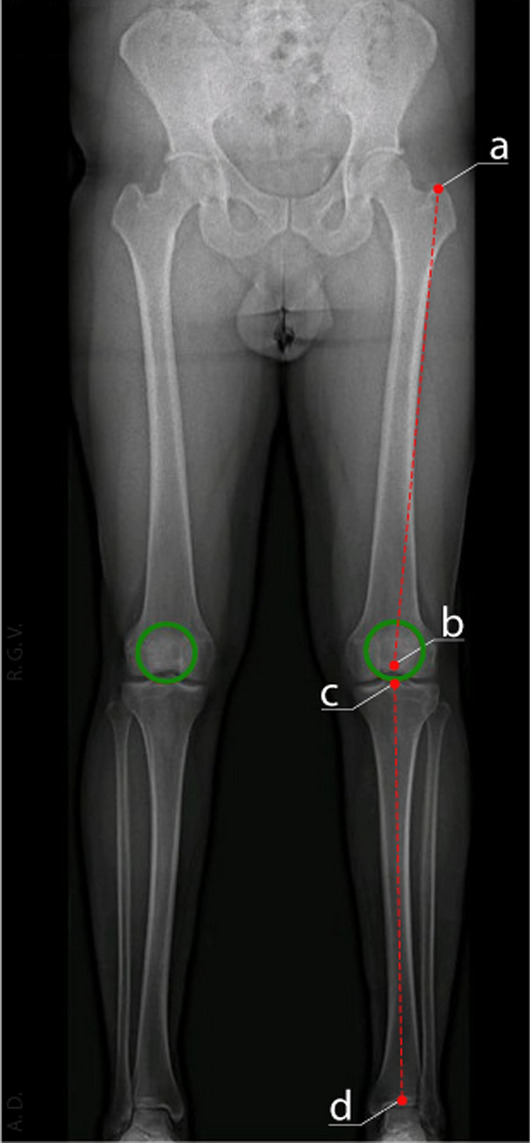
Standard full-length standing radiograph of the lower limbs illustrating correct imaging technique: the circle highlights the patella positioned at the zenith. Point “a” corresponds to the apex of the greater trochanter, point “b” to the intercondylar notch, point “c” to the intercondylar eminence, and point “d” to the center of the tibiotalar joint. Femoral length is given by the length of segment a–b, and tibial length by the length of segment c–d

Figure 2 provides an example of a full-length standing radiograph that meets all the required technical criteria, including proper inclusion of the entire lower limb and standardized patient positioning with neutral limb rotation, and illustrates the anatomical landmarks used to perform femoral and tibial length measurements.

The severity of hip osteoarthritis was classified using the Tönnis grading system, a widely accepted method for assessing degenerative changes in the hip joint. This system classifies OA from grade 0 with no signs; grade 1 with mild joint space narrowing and small osteophytes; grade 2 with moderate narrowing, cysts, and partial loss of femoral head sphericity; to grade 3 with severe narrowing, large cysts, deformity, and extensive osteophyte formation, allowing for a standardized assessment of disease progression [17].

After all radiographic measurements were completed, patients were subsequently classified as eumetric (< 10 mm difference) or dysmetric (≥ 10 mm difference) according to the calculated leg length discrepancy (LLD). No upper exclusion limit was applied, as the analysis included all patients with measurable discrepancy.

The comparison between the right and left sides was performed by subtracting the Tönnis grade of the left hip from that of the right hip. A result of 0 was classified as symmetrical, +1 as a higher grade on the right, −1 as a higher grade on the left, +2 as significantly higher on the right, and −2 as significantly higher on the left.

Comparisons were conducted among the three measurement subgroups to assess the degree of osteoarthritis in the right and left hips, as well as the asymmetry in osteoarthritis severity between the two sides.

## Statistical analysis

Statistical analyses were conducted using a combination of software tools. Data processing and comparison of parameters between the left and right sides were performed with STATA version 18.0 SE. Patients were categorized as left-side hypermetric, right-side hypermetric, or eumetric on the basis of the presence and degree of LLD. To investigate the relationship between significant LLD (≥ 10 mm) and hip degeneration, Pearson’s Chi-squared test (*χ*^2^) was applied to categorical variables. Furthermore, linear regression analysis in R version 4.4.1 was utilized to quantify the effect of LLD on OA severity.

A *p*-value < 0.05 was considered the threshold for statistical significance in all analyses, with 95% confidence intervals reported where applicable.

## Results

From an initial pool of 1672 radiographs, 220 met the inclusion criteria and were included in the final analysis. This cohort had a mean age of 72.98 years and included 142 women (64.5%) and 78 men (35.5%).

The average lengths of the tibias were 37.36 cm on the right and 37.52 cm on the left, while the lengths of the femurs, measured from the greater trochanter to the intercondylar notch, were 46.25 cm on the right side and 46.33 cm on the left side. The average total limb length was 83.61 cm on the right and 83.85 cm on the left, suggesting a mild asymmetry.

For the right hip, 40.9% of the patients presented with grade 0 OA, 51.36% with grade 1 OA, and 7.73% with grade 2 OA. The left hip presented a similar distribution, with 40% classified as grade 0, 54% as grade 1, and 5.91% as grade 2.

Among the subjects with asymmetrical OA, 23.64% showed greater severity in the right hip, while 21.82% had more severe OA on the left. Severe asymmetry, characterized by a difference of two or more degrees Tönnis, was rare, affecting only 0.45% of cases on the right and 0.91% of cases on the left.

The study classified patients into two groups according to their LLD: 173 individuals (78.64%) were considered eumetric, with discrepancies of 10 mm or less, while 47 individuals (21,36%) were dysmetric, surpassing this threshold.

Subclassifications of patients according to type of discrepancy, identifying right-sided hypermetria and left-sided hypermetria are shown in Tables 1 and 2.

**Table 1:** Analysis of tibia and femur measurements, limb comparisons, and hypermetry classifications

(A) Analysis of tibia measurements				
	Mean (cm)	St. dev.	95% CI	
Right tibia	37.36	0.22	36.92	37.80
Left tibia	37.52	0.23	37.07	37.96
∆ Right–left	−0.16	0.33	−0.22	−0.09

(B) Analysis of femur GT measurements				
	Mean (cm)	St. dev.	95% CI	
Right femur	46.25	0.24	45.77	46.73
Left femur	46.33	0.25	45.84	46.82
∆ Right–left	−0.83	0.65	−0.21	0.04

(C) Comparison of right and left lower limb				
Right tibia	37.36	Left tibia	37.52	
Right GT femur	46.25	Left GT femur	46.33	
Total sum	83.61	Total sum	83.85	
	Mean (cm)	St. Dev.	95% CI	
∆ Limb	−0.24	0.07	−0.39	−0.09

(D) Metry: −1 left hip hypermetric, 0 eumetric, +1 right hip hypermetric (cutoff 10 mm)			
	Freq.	%	Cumul.
−1	32	14.55	14.55
0	173	78.64	93.18
+1	15	6.82	100
Total	220	100	

(E) Distribution of dismetry			
	Freq.	%	Cumul.
Eumetric	173	78.64	78.64
Dysmetric	47	21.36	100
Total	220	100	

(A, B) Tibia and greater trochanter (GT) femur measurements: this section presents the mean, standard deviation (St. dev.), and 95% confidence intervals (CI) for tibia and femur GT measurements of the right and left sides, along with the difference (∆) between the two sides

(C) Comparison of right and left lower limbs: this section compares tibia and femur GT measurements between the right and left limbs, providing the mean values, standard deviations, and 95% confidence intervals for the differences (∆) between sides

(D) Hypermetry classification (−1 left hip, 0 eumetric, +1 right hip): this section categorizes participants on the basis of hip hypermetry, using a 10 mm cutoff, showing the frequency, percentage, and cumulative percentages of each category.

(E) Distribution of dysmetry: this section illustrates the frequency, percentage, and cumulative percentages of participants classified as eumetric or dysmetric

GT, greater trochanter; Freq., frequency; Cumul., cumulative

**Table 2:** Right and left hip osteoarthritis (Tönnis grade) and differences between sides

(A) right hip osteoarthritis Tönnis grade			
	Freq.	%	Cumul.
0	90	40.91	40.91
1	113	51.36	92.27
2	17	7.73	100
3	0	0	100
Total	220	100	

(B) Left hip osteoarthritis Tönnis grade			
	Freq.	%	Cumul.
0	88	40	40
1	119	54.09	94.09
2	13	5.91	100
3	0	0	100
Total	220	100	

(C) Difference in osteoarthritis right–left hips			
	Freq.	%	Cumul.
−2	2	0.91	0.91
−1	48	21.82	22.73
0	117	53.18	75.91
1	52	23.64	99.55
2	1	0.45	100
Total	220	100	

(A, B) Right and left hip osteoarthritis (Tönnis grade): this section presents the frequency, percentage, and cumulative percentages of osteoarthritis in the right and left hips, classified by Tönnis grade (0–3)

(C) Difference in osteoarthritis between right and left hips: this section displays the frequency, percentage, and cumulative percentages of differences in osteoarthritis grades between the right and left hips

Freq., frequency; Cumul., cumulative

The Pearson Chi-squared test revealed no significant dependence between osteoarthritis and discrepancy for the right hip (*p* = 0.520) or the left hip (*p* = 0.185). However, a statistically significant dependence was found between the variables of discrepancy and the difference in osteoarthritis between the right and left sides (*p* = 0.002), as shown in Table 3.

**Table 3:** Multivariate analysis of hypermetry and osteoarthritis

(A) Multivariate analysis: hypermetria—∆ osteoarthritis						
Hypermetria		∆ Osteoarthritis right–left				
		−2	−1	0	1	2
	> Left	0	8	12	11	1
	0	1	33	102	37	0
	> Right	1	7	3	4	0
	Total	2	48	117	52	1
	Pearson *χ*^2^(8) = 24.50, *p* = 0.002					

(B) Multivariate analysis: hypermetria—right hip osteoarthritis (Tönnis grade)						
Hypermetria		Right hip osteoarthritis (Tönnis grade)				
		0	1	2	3	Total
	> Left	10	18	4	0	32
	0	73	87	13	0	173
	> Right	7	8	0	0	15
	Total	90	113	17	0	220
	Pearson *χ*^2^(4) = 3.23, *p* = 0.520					

(C) Multivariate analysis: hypermetria—left hip osteoarthritis (Tönnis grade)						
Hypermetria		Left hip osteoarthritis (Tönnis grade)				
		0	1	2	3	Total
	> Left	12	19	1	0	32
	0	71	93	9	0	173
	> Right	5	7	3	0	15
	Total	88	119	13	0	220
	Pearson *χ*^2^(4) = 6.19, *p* = 0.185					

This table presents the association between hypermetry categories and osteoarthritis grades for both the right (A) and left (B) hips, as well as the difference in osteoarthritis grades between the two sides (C). Statistical analysis includes Pearson’s Chi-squared test (*χ*^2^) and *p*-values

**Fig. 3:**
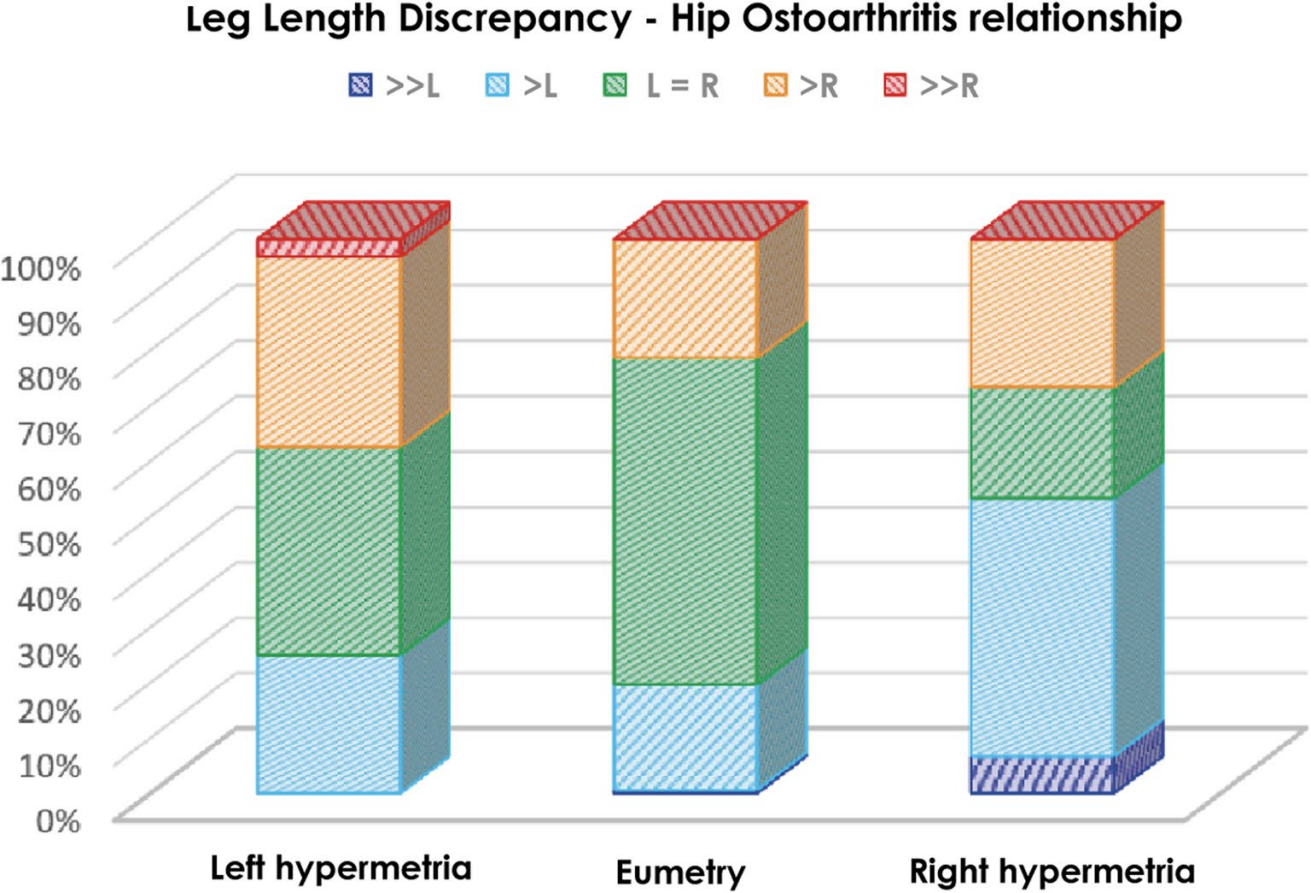
Comparison of prevalence of osteoarthritis between hypometric and hypermetric limb. “>” and “> >” indicate increasing differences in Tönnis grade between the two hips, while “L = R” indicates symmetrical osteoarthritis

The analysis indicated a greater prevalence of osteoarthritis in the hypometric limb. The data, presented in Fig. 3, demonstrated that in the presence of left-sided hypermetria, there is not only a lower prevalence of severe osteoarthritis on the left side but also an increase in cases of more severe osteoarthritis on the right side. In contrast, eumetric patients exhibited a more uniform distribution of coxarthrosis between the two limb.

## Discussion

The most important finding of this study is that there is a statistically significant correlation between leg length discrepancy (LLD) using a cutoff of 10 mm and lower limb osteoarthritis. Specifically, our data highlight how LLD may be associated with an asymmetrical distribution of hip osteoarthritis, with a higher incidence in the shorter limb compared with the hypermetric limb.

These findings confirm that, as the LLD increases, the degree of osteoarthritis tends to be more severe. Literature has already demonstrated that LLD greater than 20 mm can alter gait and the biomechanics of walking, changing the load-bearing cartilage areas [9].

The 10-mm threshold used in this study is consistent with previous literature indicating that discrepancies above this value are more likely to alter gait biomechanics and pelvic alignment, potentially leading to asymmetric joint loading and degenerative changes [4]. Conversely, differences below approximately 10 mm are generally asymptomatic and within the physiological range of joint compensation [2].

However, to the authors’ knowledge, no studies in literature have specifically selected patients using the criteria used for this study: namely, a cutoff of 10 mm, measured from the apex of the greater trochanter (GT) in an upright position; patients older than 65 years; and exclusion of patients with prosthetic implants or fixation devices.

In most studies, measurements are taken from the femoral head, in supine patients, or without accounting for the presence of prosthetic implants, introducing potential biases in measurements [4–7, 16].

Liu et al. used a 10 mm cutoff for LLD in a radiographic evaluation of over 600 human specimens and they did not identify a strong correlation between moderate LLD and hip arthritis [16]. Conversely, Kim et al. found an increasing correlation between hypermetry of one limb and contralateral hip arthritis, particularly when the LLD was ≥ 2 cm [4]. Murray et al. examined 255 patients aged 18–70 years, analyzing the correlation between LLD (≥ 0.5 cm) and osteoarthritis of the hip, L4–L5, and L5–S1 vertebrae [5].

Their analysis showed significant validity only for the male subgroup but not for the female subgroup. Golightly et al. used a 2 cm cutoff for LLD and found a greater severity of contralateral coxarthrosis in unadjusted analyses compared with the hypermetric side [6].

However, this correlation did not reach statistical significance in multiple logistic regression models, with a *p*-value of 0.07. On the contrary, Tallroth et al., in a study with 100 patients, found a higher incidence of osteoarthritis in the hypermetric limb compared with the contralateral side [7].

These results underscore the complex picture presented in literature: while it is now clear that LLD can increase the risk of arthritic degeneration at various levels (L4–L5, L5–S1, hip, and knee), the available data are often conflicting, especially regarding the significant cutoffs limits and measurement methods.

The strengths of this study lie in the careful selection of patients and methods for measuring lower limb discrepancies. Non-suitable radiographs were excluded, and only patients over 65 years old were selected, thereby excluding younger patients who might not yet have developed degenerative processes, reducing potential biases. Moreover, the exclusion of patients with prosthetic implants or fixation devices allowed us to avoid interference in axis and measurement assessments. Specifically, limb length discrepancies were derived from bilateral measurements of the femur and tibia using standardized anatomical landmarks, from the greater trochanter proximally to the knee and ankle landmarks distally, providing a reliable assessment of limb asymmetry in patients in a weight-bearing standing position. The use of calibrated digital radiographs and standardized PACS (Picture Archiving and Communication System) measurement tools, together with image calibration verification and repeated bilateral measurements, further improves the reliability and reproducibility of the measurement process.

Some of the aforementioned studies, in fact, take measurements in supine patients (e.g., Golightly et al., Liu et al.), which may be more biased owing to possible hip or knee flexion [6, 18].

Other studies reference the upper margin of the femoral head, which may be affected by arthritic degeneration and thus compromise results (e.g., Kim et al., Murray et al.) [4, 5].

This study presents some limitations that need to be highlighted. First, the study setting presents the intrinsic limitations of retrospective design. Second, the sample size is relatively small and the selection of patients from a hospital database could represent a selection bias, potentially not being representative of the general population. A multicenter study with a larger and more diverse cohort could help generalize the findings more effectively. Third, the exclusion of patients with prosthetic implants could constitute another bias, as it excludes patients with a more severe degree of osteoarthritis in the past. Fourth, patients were classified as eumetric or hypermetric regardless of potential symptomatology, whereas LLD is often asymptomatic and sometimes not recognized by the patients themselves. The 10-mm threshold was chosen a priori for its clinical and biomechanical relevance, but no standardized classification of mild, moderate, or severe LLD exists. Previous studies used variable cutoffs (5–20 mm) depending on design and population [4–6, 18].

No upper exclusion limit was set, as the analysis aimed to include all discrepancies ≥ 10 mm that may be clinically meaningful. Future research should integrate functional assessments, symptom reporting, and gait analysis to better correlate LLD with clinical outcomes. Fifth, radiographic measurements, even if accurately selected from standing radiographs, may be influenced by slight variations in patient positioning or minor imaging inaccuracies. Furthermore, relying solely on radiographs without incorporating clinical examinations or advanced imaging techniques, such as magnetic resonance imaging (MRI), could lead to an incomplete understanding of joint degeneration. Sixth, the study did not take into account factors such as body mass index (BMI), activity levels, or comorbidities, which could influence the progression of hip osteoarthritis. The inclusion of these variables in future analyses would provide a more nuanced and accurate interpretation of the data. Seventh, focusing on patients aged 65 years or older highlights the risks of osteoarthritis, but limits the applicability to younger individuals. Expanding the age range in future research would provide a broader perspective on the effects of limb length discrepancy at different life stages. Eighth, although this study focused on X-rays of whole limbs, from pelvis to ankle, we did not directly evaluate spinopelvic or gait parameters. Given that compensatory adaptations resulting from LLD occur predominantly in the coronal plane, affecting pelvic obliquity, spinal alignment, and lower limb loading, future studies integrating gait analysis and spinopelvic alignment assessment may provide deeper insight into the biomechanical chain linking LLD to joint degeneration.

Despite these limitations, this study offers valuable insights into the management of limb length discrepancies (LLD) in various age groups and outlines potential therapeutic strategies to address them. In pediatric patients, LLD is commonly observed, especially following trauma. In mild cases, especially those accompanied by symptoms, conservative treatments, such as the use of heel lift insoles, can provide effective relief. However, in more severe cases, surgical interventions such as epiphysiodesis may be an effective method to correct discrepancies and promote balanced limb growth. For adults without arthritis, the management of LLD presents a different challenge. The study raises the question of whether conservative treatments, such as insoles, can play a preventive role.

In adults with arthritis, the management of LLD requires meticulous preoperative planning to avoid creating discrepancies during prosthetic replacement surgery [17, 18]. Key measures include careful preoperative planning, the use of intraoperative techniques and, potentially, the use of advanced tools such as intraoperative navigation. These strategies are essential to prevent LLD and ensure better outcomes with prosthetic replacements [19, 20].

When LLD occurs as a consequence of prosthetic surgery (iatrogenic LLD), conservative treatments such as heel lift insoles can alleviate symptoms and restore functional alignment [17]. However, each case must be assessed individually to determine whether these measures are sufficient or whether further surgery is required to achieve optimal results.

## Conclusions

This study shows a statistically significant association between lower limb discrepancy (LLD) of ≥ 10 mm and the development of contralateral hip osteoarthritis (OA) in patients aged 65 years or older. These data indicate that moderate LLD may alter gait biomechanics, leading to asymmetrical joint loading and accelerated degenerative changes in the contralateral hip. These results emphasize the importance of early LLD detection and tailored interventions to prevent or mitigate joint degeneration. From pediatric to elderly populations, addressing LLD with preventive measures, conservative treatments, or surgical solutions can improve outcomes. Furthermore, meticulous preoperative planning is essential to avoid iatrogenic LLD during procedures such as total hip arthroplasty (THA). Future research should expand on these findings, exploring LLD management across different age groups and incorporating functional assessments for a more comprehensive understanding.

## Data Availability

A paper copy of the database is available at Città della salute e della Scienza di Torino.
